# Temporal Patterns of Multimorbidity Progression Among Inpatients With a Recorded Hypertension Diagnosis Across Three Counties in Rural Henan, China: Retrospective Cohort Study Using Temporal Network Analysis

**DOI:** 10.2196/93217

**Published:** 2026-10-23

**Authors:** Yifei Wang, Yanna Mao, Xi Wang, Rongmei Liu, Manman Cui, Ruihua Feng

**Affiliations:** 1 Institute of Medical Information, Chinese Academy of Medical Sciences and Peking Union Medical College, No. 3 Yabao Road, Chaoyang District, Beijing, 100020, China; 2 School of Health Management, Southern Medical University, Guangzhou, Guangdong, China; 3 Henan Key Laboratory for Health Management of Chronic Diseases, Central China Fuwai Hospital, Central China Fuwai Hospital of Zhengzhou University, Zhengzhou, Henan, China; 4 People's Medical Publishing House, Beijing, China

**Keywords:** hypertension, multimorbidity, temporal network analysis, disease progression, rural population

## Abstract

**Background:**

Hypertension is a key driver of multimorbidity; however, its role in shaping dynamic disease progression, especially in rural populations, remains underexplored.

**Objective:**

This study aimed to characterize temporal patterns of multimorbidity progression among inpatients with a recorded hypertension diagnosis and to examine whether these patterns differed by sex, age, insurance type, and county.

**Methods:**

We analyzed electronic health record data from 34,099 inpatients with a recorded hypertension diagnosis across 3 rural counties in Henan, China (2019‐2023), constructing a temporal comorbidity network of 250 disease nodes and 1512 disease transitions. Statistical validation was performed using bootstrap resampling, case-dropping, and permutation tests. Subgroup analyses were conducted by sex, age, insurance type, and county.

**Results:**

The temporal comorbidity network revealed a densely interconnected core involving hypertension, atherosclerotic heart disease, and cerebral infarction, with type 2 diabetes occupying a bridging position between cardiovascular and multisystem disease clusters. The ordering of dominant recorded pathways differed by sex (earlier coronary involvement among female inpatients vs more direct progression to stroke among male inpatients), was more complex in inpatients older than 70 years of age, and was more diffuse among those covered by urban-rural resident insurance compared with urban employee insurance.

**Conclusions:**

Multimorbidity among inpatients with recorded hypertension followed structured and heterogeneous temporal patterns that differed by sex, age, insurance type, and county. Cardiovascular and cerebrovascular conditions formed the principal recorded progression pathways, while type 2 diabetes connected cardiovascular pathways with broader multisystem complications. These findings support further evaluation of risk-stratified and locally adapted approaches to chronic disease prevention and longitudinal care.

## Introduction

Hypertension is a major modifiable risk factor for cardiovascular diseases (CVDs) and a dominant contributor to global multimorbidity. In China, over 270 million individuals live with hypertension, with the burden particularly concentrated in rural areas where health care infrastructure, health literacy, and early screening are limited [1]. This condition rarely occurs in isolation. Instead, it often initiates a cascade of comorbidities, including stroke, ischemic heart disease, diabetes, and chronic kidney disease, leading to premature mortality, disability, and catastrophic health expenditures [2]. Multimorbidity, typically defined as the presence of 2 or more chronic conditions, has become a central challenge in population health and health system sustainability. In the European Union alone, over 50 million people are affected, and projections suggest that complex multimorbidity (≥4 conditions) will increase from 10% in 2015 to 17% by 2035 [3]. The implications are profound: multimorbidity is associated with reduced quality of life, functional decline, and substantial health care costs [3].

Despite its significance, most research on multimorbidity has employed cross-sectional approaches, capturing only static disease clustering rather than dynamic disease progression. This limits the ability to identify early transitions, turning points, or critical intervention windows [4]. In recent years, there has been a shift toward longitudinal frameworks and data-driven disease trajectory models that can track the accumulation, ordering, and evolution of chronic conditions over time [4,5]. These include techniques such as trajectory modeling, latent class analysis, and, increasingly, temporal network analysis (TNA), which conceptualizes disease interactions as evolving networks [6]. Hypertension is recognized as a key initiating node in these networks. Longitudinal simulations have shown that individuals with hypertension have significantly higher lifetime risks of progressing to diabetes, heart failure, and cerebrovascular events, with variability by age, sex, and socioeconomic status [4,7]. In particular, trajectory simulations from Chinese population data demonstrate that hypertension precedes and predicts a broad spectrum of multimorbidity profiles, underscoring its centrality in disease evolution [7].

Hypertension frequently coexists with cardiovascular, metabolic, cerebrovascular, and kidney-related conditions, and the burden of multimorbidity substantially increases with age [8]. Previous studies have documented the prevalence, clustering, and clinical consequences of multimorbidity among people with hypertension across different populations [9]. Longitudinal investigations have further shown that multimorbidity dynamically evolves through heterogeneous sequences of disease emergence and accumulation, rather than as a simple increase in disease counts [10]. However, existing research has largely focused on prevalence, cross-sectional co-occurrence, or static clustering patterns, and relatively few studies have examined the temporal ordering of diagnoses across repeated health care encounters [11,12]. Characterizing these temporal patterns may complement conventional measures by revealing which conditions are repeatedly recorded earlier or later in longitudinal clinical records and whether these patterns vary across population subgroups.

However, most trajectory research has been conducted in high-income countries [4]. There is a critical lack of evidence from low- and middle-income settings, especially in rural counties of China, where occupational exposures (eg, pesticides), local dietary customs, and unequal health resource allocation create unique risk structures [3,4]. Furthermore, within-country heterogeneity—between counties, economic strata, and health insurance groups—has been underexplored.

To address this evidence gap, this study aimed to characterize temporal patterns of multimorbidity progression among inpatients with a recorded diagnosis of hypertension and to examine whether these patterns differed by sex, age, insurance type, and county across 3 socioeconomically diverse counties in Henan Province, China. We used TNA to reconstruct statistically supported, temporally ordered disease transitions from longitudinal inpatient records. By identifying common progression structures and subgroup-specific patterns, the study sought to provide evidence to support more risk-stratified and context-sensitive approaches to chronic disease prevention and management in underserved rural populations.

## Methods

### Data Source and Study Design

This retrospective observational study was conducted using inpatient electronic health records (EHRs) collected from 6 local hospitals across 3 counties in Henan Province, China, covering the period from January 1, 2019, to December 31, 2023. The dataset comprised 300,241 inpatient visits, including inpatient identifiers, admission dates, discharge diagnoses, and *ICD-10* (*International Classification of Diseases, 10th Revision*) codes.

Hypertension was treated as the index condition, given its recognized role as a central initiating disease in multimorbidity trajectories [1,12]. The present analysis was based on inpatient EHR data because these records provided patient-linked diagnostic information across repeated hospital admissions, allowing the temporal ordering of recorded chronic conditions to be reconstructed. Hypertension was used as a cohort-defining recorded diagnosis and was not necessarily the primary reason for hospitalization. Accordingly, the study was designed to characterize temporal multimorbidity patterns among hospitalized patients with a recorded hypertension diagnosis rather than to represent all individuals with hypertension in the underlying county populations. The study design followed a population-level, longitudinal disease trajectory framework, consistent with established approaches in multimorbidity trajectory research and temporal disease network construction [3-5]. This ensures that the evolution of comorbidity patterns analyzed thereafter accurately reflects the disease progression in hypertensive inpatients. Because this study used the complete set of eligible inpatient EHR records available from the 6 participating hospitals rather than data obtained through a probability-based survey design, survey sampling weights were not applicable. All analyses were therefore conducted using the unweighted analytical cohort. This study was reported in accordance with the STROBE (Strengthening the Reporting of Observational Studies in Epidemiology; Checklist 1) statement for cohort studies and the RECORD (Reporting of Studies Conducted Using Observational Routinely Collected Health Data; Checklist 2) statement.

### Ethical Considerations

This study was approved by the Ethics Committee of Research and New Technology/New Business of Fuwai Central China Cardiovascular Hospital (approval number 2025; ethics review number 25). Owing to the retrospective study design and the use of routinely collected inpatient EHR data, the requirement for individual informed consent was waived by the ethics committee.

The source data received by the research team contained direct inpatient identifiers, including names and ID numbers. Before the construction of the analytical dataset, these direct identifiers were removed and replaced with encrypted inpatient identifiers, which were used solely to link multiple inpatient admissions belonging to the same inpatient. All statistical analyses were conducted using the resulting deidentified analytical dataset, and no directly identifying information was included in the reported results.

Because this study involved the secondary analysis of existing inpatient records and no inpatients were directly recruited or contacted, participant compensation was not applicable.

### Data Preprocessing

Raw EHR data underwent standardized preprocessing procedures to ensure data integrity and analytical reproducibility. Specifically, this procedure included inpatient identity harmonization, diagnostic code standardization, temporal normalization, and record exclusion. Encrypted inpatient identifiers were harmonized across records to enable the longitudinal linkage of multiple admissions belonging to the same inpatient. All diagnoses were mapped to the National Clinical *ICD-10* version 2.0 standard, following recommended practices for multimorbidity research [2,3]. Admission dates were converted to a unified format (“yyyy-mm-dd”) to enable precise temporal ordering. A total of 4300 inpatient records were excluded due to missing critical fields (patient ID, diagnosis code, or admission date), consistent with data quality control strategies in large-scale EHR studies [4]. No imputation was applied to missing diagnostic or temporal variables, as incomplete records could compromise disease sequencing accuracy.

### Cohort Selection Criteria

Cohort selection was carried out according to predefined inclusion and exclusion criteria. To ensure that the comorbidity patterns analyzed would accurately reflect the disease progression of hypertensive inpatients, the inclusion criteria were (1) inpatients with at least 2 inpatient records during the study period, (2) each inpatient record must include at least 2 diagnoses, (3) at least one diagnosis classified as a CVD, and (4) at least one admission containing a hypertension diagnosis (*ICD-10*: I10.x). The exclusion criteria were (1) inpatients with only one inpatient record; (2) inpatients without any hypertension diagnosis; (3) records with missing key identifiers or temporal information such as inpatient ID, diagnosis, or date; and (4) duplicate inpatient records. After applying the predefined inclusion and exclusion criteria, 34,099 inpatients with at least one recorded hypertension diagnosis during the study period were included in the final analytical cohort.

### Baseline Comorbidity Pattern Analysis

To characterize coexisting disease patterns at cohort entry, chronic conditions recorded during each inpatient’s first admission in the study dataset were aggregated into inpatient-level transactions. The number of distinct chronic conditions was calculated after deduplicating diagnosis codes within the cohort-entry admission, with hypertension included in the condition count. Frequent disease combinations were identified using the Apriori algorithm with a minimum support threshold of 0.01. Itemset length was not restricted during the mining process; for readability, only the top-ranked 2-, 3-, and 4-itemsets are presented in Table 1.

**Table 1.** Top-ranked baseline disease combinations identified by association rule mining among 34,099 inpatients with at least one recorded diagnosis of hypertension across 6 hospitals in 3 counties in Henan Province, China, 2019‐2023, based on chronic conditions recorded during each inpatient’s first admission (support ≥0.01).^a^

Length and *ICD-10*^b^ code	Itemsets	Support
2-itemsets		
I25.1, I10.X	Atherosclerotic heart disease and hypertension	0.27
I63.9, I10.X	Cerebral infarction and hypertension	0.25
I63.9, I25.1	Cerebral infarction and atherosclerotic heart disease	0.13
I50.9, I25.1	Heart failure and atherosclerotic heart disease	0.12
I50.9, I10.X	Heart failure and hypertension	0.10
3-itemsets		
I25.1, I63.9, I10.X	Atherosclerotic heart disease, cerebral infarction, and hypertension	0.10
I25.1, I50.9, I10.X	Atherosclerotic heart disease, heart failure, and hypertension	0.08
I50.9, I25.1, I25.5	Heart failure, atherosclerotic heart disease, and ischemic cardiomyopathy	0.04
I25.1, I10.X, E11.9	Atherosclerotic heart disease, hypertension, and type 2 diabetes	0.03
I63.9, I10.X, E11.9	Cerebral infarction, hypertension, and type 2 diabetes	0.03
4-itemsets		
I25.1, I50.9, I10.X, I25.5	Atherosclerotic heart disease, heart failure, hypertension, and ischemic cardiomyopathy	0.03
I25.1, I50.9, I63.9, I10.X	Atherosclerotic heart disease, heart failure, cerebral infarction, and hypertension	0.02
I25.1, I50.9, I10.X, E11.9	Atherosclerotic heart disease, heart failure, hypertension, and type 2 diabetes	0.01
I25.1, I63.9, I10.X, E11.9	Atherosclerotic heart disease, cerebral infarction, hypertension, and type 2 diabetes	0.01
I50.9, I63.9, I25.1, I25.5	Heart failure, cerebral infarction, atherosclerotic heart disease, and ischemic cardiomyopathy	0.01

a Only the top-ranked 2-, 3-, and 4-itemsets are displayed for readability; itemset presentation was not used as an inpatient-level exclusion criterion.

b *ICD-10*: *International Classification of Diseases, 10th Revision*.

### Construction of the Temporal Comorbidity Network

To characterize statistically robust disease progression patterns, we constructed a temporal comorbidity network (TCN)—a weighted, directed network representing population-level disease evolution, following established disease trajectory modeling frameworks [5,6,12,13]. In the TCN, nodes represent distinct *ICD-10* disease categories, and directed edges represent statistically significant temporal transitions between diseases. Across the cohort, 24,959 unique, temporally ordered disease diagnosis pairs were identified and used as candidate temporal comorbidity pairs for statistical inference.

### Network Construction Procedure

For each inpatient, chronic disease records were chronologically ordered according to admission dates. Only the earliest recorded occurrence of each disease within the study dataset was retained, and subsequent records of the same disease were excluded to reduce repeated-counting bias associated with recurrent hospitalizations. The date assigned to each disease, therefore, represents its first recorded occurrence in the available inpatient EHR data rather than the true date of disease onset or initial clinical diagnosis.

The retained disease records were then grouped by inpatient admission to generate an inpatient-specific longitudinal sequence of effective admission time points. Conditions first recorded during the same admission were assigned to the same time point, and no temporal direction was inferred between them. For each pair of consecutive effective admissions, all eligible disease pairs were generated by linking a disease first recorded at admission *t* to a different disease first recorded at the immediately subsequent admission *t*+1. Thus, each inpatient contributed a sequence of temporally ordered disease pairs derived from successive inpatient admissions.

As a hypothetical example, consider an inpatient with hypertension (I10.X) and atherosclerotic heart disease (I25.1) first recorded during admission 1, type 2 diabetes (E11.9) first recorded during admission 2, and cerebral infarction (I63.9) first recorded during admission 3. Because hypertension and atherosclerotic heart disease were first recorded during the same admission, no temporal direction was assigned between them. The consecutive-admission procedure generated the pairs I10.X→E11.9 and I25.1→E11.9 between admissions 1 and 2, and E11.9→I63.9 between admissions 2 and 3. Repeated recordings of previously observed conditions during later admissions were ignored. This hypothetical example is provided solely to illustrate the sequence-generation procedure and does not represent an actual patient record.

Patient-level disease pairs were subsequently aggregated across the cohort. A chi-square test was first used to determine whether the observed co-occurrence frequency of each disease pair exceeded that expected under independence. A significance level of α=.05 was used, and pairs with *P*<.05 were retained. For each retained pair, a binomial test was then used to determine whether one temporal direction occurred more frequently than the reverse direction under a null probability of 0.5. Pairs with *P*<.05 in both tests were represented as directed edges in the population-level TCN, with edge weights corresponding to the observed frequency of the temporal transition among inpatients.

### Robustness and Validation Analysis

To evaluate the stability and reliability of the inferred TCN, 3 complementary robustness analyses were conducted, following best practices in network epidemiology [3,5]. First, bootstrap resampling with replacement was performed 1000 times. The TCN was reconstructed in each bootstrap sample, and the proportion of bootstrap networks in which each edge reappeared was calculated. Edges reproduced in at least 95% of the bootstrap samples were retained to form the Stable Network, representing the most reproducible core subnetwork of the original TCN.

Second, a case-dropping sensitivity analysis was conducted to assess the robustness of the network to changes in cohort composition. In each of the iterations, 3410 of 34,099 (10.0%) inpatients were randomly excluded, and the TCN was reconstructed. Similarity between the edge sets of each reduced network and the original TCN was quantified using the Jaccard similarity coefficient.

Third, a permutation-based null-network analysis was performed to determine whether the observed network topology exceeded that expected by chance. The temporal order of recorded disease diagnoses was randomly permuted for each inpatient, and the TCN was reconstructed using the same analytical procedure for each of the 1000 permutations. The number of nodes and edges, network density, and average clustering coefficient of the original TCN were compared with the corresponding null distributions.

For clarity, the original TCN refers to the full network constructed from all statistically significant temporal disease transitions identified in the observed cohort. The Stable Network is a bootstrap-derived subnetwork of the original TCN comprising edges reproduced in at least 95% of the bootstrap samples. The permutation-based null model refers to the distribution of randomized networks generated by disrupting the observed temporal ordering of diagnoses and serves as a chance-expectation benchmark for the original TCN.

## Results

After applying the predefined eligibility criteria, 34,099 inpatients with at least 1 recorded hypertension diagnosis during the study period were included in the final analytical cohort (Figure 1). Baseline demographic and clinical characteristics were derived from each inpatient’s first inpatient record in the study dataset (Table 2). The cohort included 17,071 (50.1%) male inpatients and 17,028 (49.9%) female inpatients.

**Figure 1.**
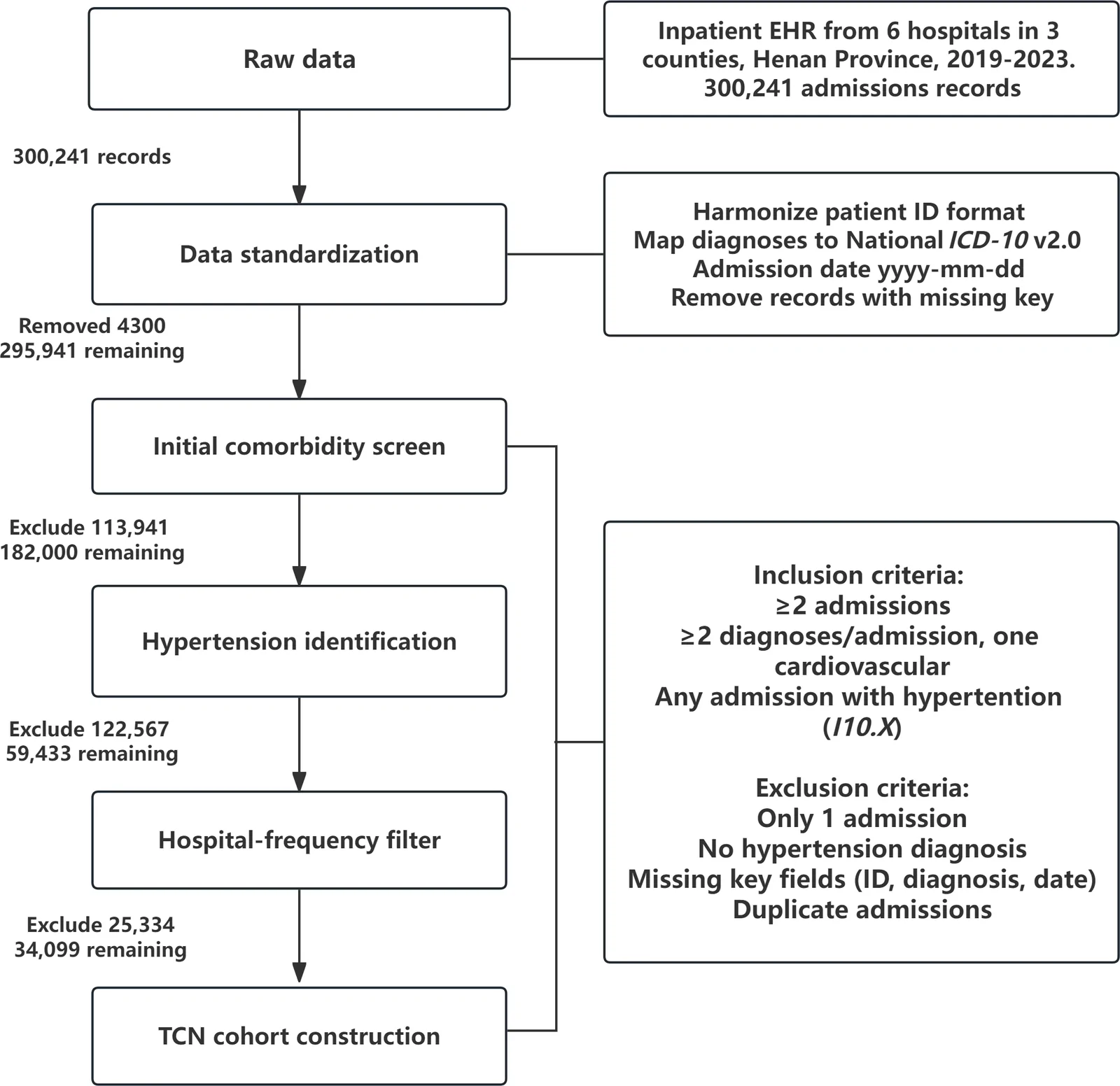
Flowchart of inpatient record processing and inpatient selection for this retrospective cohort of inpatients with at least one recorded hypertension diagnosis across 6 hospitals in 3 counties in Henan Province, China, 2019‐2023. EHR: electronic health records; *ICD-10*: *International Classification of Diseases, 10th Revision*; TCN: temporal comorbidity network.

**Table 2.** Baseline demographic and clinical characteristics of 34,099 inpatients with at least one recorded diagnosis of hypertension across 6 hospitals in 3 counties in Henan Province, China, 2019‐2023, based on each inpatient’s first record in the study dataset.

Characteristic	Total (N=34,099)	Male (n=17,071)	Female (n=17,028)	SMD^a^
Age group (y), n (%)				0.096
<30	77 (0.2)	54 (0.3)	23 (0.1)	
30‐39	424 (1.2)	277 (1.6)	147 (0.9)	
40‐49	2101 (6.2)	1154 (6.8)	947 (5.6)	
50‐59	6659 (19.5)	3337 (19.5)	3322 (19.5)	
60‐69	9848 (28.9)	4809 (28.2)	5039 (29.6)	
≥70	14,990 (44.0)	7440 (43.6)	7550 (44.3)	
County, n (%)				0.057
Jia County	21,305 (62.5)	10,866 (63.7)	10,439 (61.3)	
Yiyang County	10,047 (29.5)	4808 (28.2)	5239 (30.8)	
Mianchi County	2747 (8.1)	1397 (8.2)	1350 (7.9)	
Insurance type, n (%)				0.375
Urban Resident Basic Medical Insurance	27,975 (82.0)	12,988 (76.1)	14,987 (88.0)	
Urban Employee Basic Medical Insurance	3514 (10.3)	2718 (15.9)	796 (4.7)	
Other payment	2610 (7.7)	1365 (8.0)	1245 (7.3)	

a SMD: standardized mean difference.

Patients aged 70 years or older constituted the largest age group (14,990/34,099, 44.0%), followed by those aged 60 to 69 years (9848/34,099, 28.9%). The standardized mean difference in age-group distribution between male and female inpatients was 0.096.

Geographically, 62.5% (21,305/34,099) of inpatients resided in Jia County, 29.5% (10,047/34,099) in Yiyang County, and 8.1% (2747/34,099) in Mianchi County. Health insurance data indicated that 82.0% (27,975/34,099) of inpatients were covered by Urban and Rural Resident Basic Medical Insurance, with a higher proportion of women (14,987/17,028, 88.0%) than men (12,988/17,071, 76.1%) under this scheme (standardized mean difference=0.375). Conversely, a greater proportion of men (2718/17,071, 15.9%) were covered by Urban Employee Insurance than women (796/17,028, 4.7%).

Based on the distinct chronic conditions recorded during each patient’s first inpatient admission in the study dataset, out of 34,099 inpatients, 4499 (13.2%) had one recorded chronic condition, 9777 (28.7%) had two, 9402 (27.6%) had three, 5769 (16.9%) had four, and 4652 (13.6%) had five or more recorded chronic conditions. Overall, 29,600 (86.8%) inpatients had at least 2 recorded chronic conditions at cohort entry.

All 34,099 inpatients contributed at least 2 effective inpatient admission time points, with only the earliest recorded occurrence of each chronic disease retained. Of these inpatients, 27,381 (80.3%) contributed 2 effective time points and 6718 (19.7%) contributed 3 or more, with a range of 2 to 11 time points per inpatient. The median interval between each inpatient’s first and last effective admission was 738 (IQR 526‐856) days.

Collectively, the inpatient-specific longitudinal sequences consisted of 44,524 intervals between consecutive effective admissions. Disease pairs were generated from these consecutive intervals and aggregated at the population level, yielding 24,959 unique candidate temporally ordered disease pairs for statistical testing. These candidate pairs formed the longitudinal basis for the construction of the TCN.

Table 1 displays only the top-ranked 2-, 3-, and 4-itemsets for readability. This presentation does not indicate that inpatients with more than 4 recorded chronic conditions were excluded. At cohort entry, 4652 out of 34,099 (13.64%) inpatients had 5 or more recorded chronic conditions, and these inpatients were retained in the subsequent analyses. The TCN was constructed from pairwise, temporally ordered disease transitions and therefore incorporated eligible transitions irrespective of the total number of chronic conditions recorded for an individual inpatient.

Following statistical testing of the 24,959 candidate temporally ordered disease pairs, the original TCN comprised 250 disease nodes and 1512 directed edges. Of the 1512 edges in the original TCN, 682 (45.1%) were reproduced in at least 95% of the 1000 bootstrap samples and were retained in the Stable Network. The Stable Network comprised 189 nodes and represented the most reproducible core subnetwork of the original TCN. The 682 stable edges accounted for 2.73% of all 24,959 candidate temporal disease pairs.

In the case-dropping sensitivity analysis, the mean Jaccard similarity between the edge sets of the reduced networks and the original TCN was 0.8587 (SD 0.0075) across 1000 iterations, with a coefficient of variation below 1%, indicating limited sensitivity to the random exclusion of 10% of the cohort.

The permutation-based null model generated networks with a mean of 117.50 (SD 6.62) nodes and 134.30 (SD 11.51) edges. Compared with the null-network distributions, the original TCN had greater network density (0.0243 vs mean 0.0098, SD 0.0008) and a substantially higher average clustering coefficient (0.6557 vs mean 0.0124, SD 0.0108). The clustering coefficient of the original TCN exceeded the null mean by more than 50 SDs, supporting the conclusion that the observed network structure was unlikely to have arisen from random temporal ordering (Table 3).

**Table 3.** Robustness validation and topological characteristics of the original temporal comorbidity network (TCN), its bootstrap-derived Stable Network, and permutation-based null networks among 34,099 inpatients with at least one recorded hypertension diagnosis across 6 hospitals in 3 counties in Henan Province, China, 2019‐2023.^a^

Metric	Original TCN	Stable Network	Null networks, mean (SD)
Number of nodes	250	189	117.50 (6.62)
Number of edges	1512	682	134.30 (11.51)
Network density	0.0243	0.0192	0.0098 (0.0008)
Average clustering coefficient	0.6557	0.5791	0.0124 (0.0108)

a The original TCN comprised all statistically significant temporal disease transitions identified in the observed cohort. The Stable Network was a subnetwork of the original TCN containing edges reproduced in at least 95% of 1000 bootstrap samples. Null-network values are the mean and SD from 1000 networks generated by randomly permuting the temporal order of recorded diagnoses within each inpatient.

The original TCN exhibited a hub-and-spoke structure, with hypertension (I10.X), atherosclerotic heart disease (I25.1), cerebral infarction (I63.9), and heart failure (I50.9) occupying central positions. The most prominent directed pathways originating from hypertension were those to atherosclerotic heart disease and to cerebral infarction. Hypertension, atherosclerotic heart disease, and cerebral infarction also formed a densely interconnected region within the network (Figure 2).

**Figure 2.**
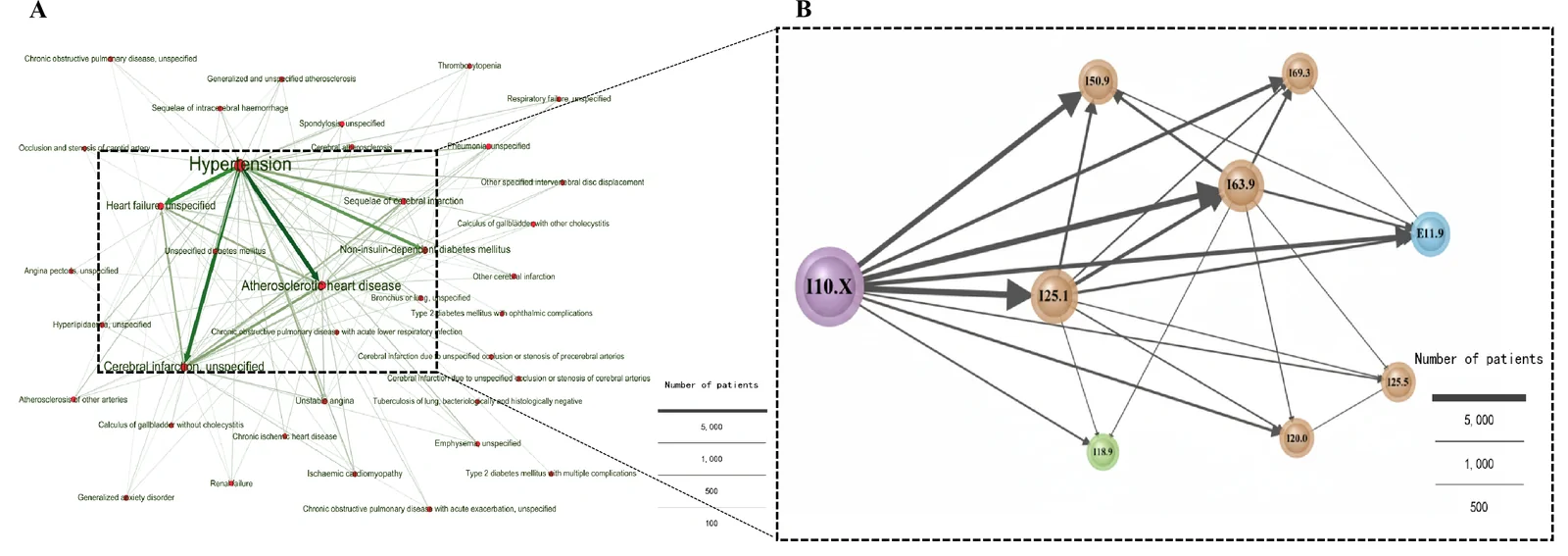
(A) Population-level temporal comorbidity network among 34,099 inpatients with at least one recorded diagnosis of hypertension across 6 hospitals in 3 counties in Henan Province, China, 2019‐2023, with an enlarged view of the densely connected core region of (B) core comorbidity transition network.

Type 2 diabetes (E11.9) occupied a bridging position between cardiovascular and metabolic disease clusters. Recorded transitions connected type 2 diabetes with hypertension and diabetes-related renal, neurological, and ophthalmic complications, including E11.4, E11.7, and E11.3. This network position indicates that type 2 diabetes connected otherwise distinct groups of recorded cardiovascular and multisystem conditions.

Additional recorded transitions from hypertension included carotid artery stenosis and systemic atherosclerosis. Downstream nodes in the network included chronic obstructive pulmonary disease (J44.0), emphysema (J43.9), renal failure (N18.9), and sequelae of cerebrovascular disease (I69.X). Renal failure and cerebrovascular sequelae received directed edges from multiple upstream nodes, indicating that they occurred as common downstream recorded conditions in several network pathways.

Subgroup-specific TCNs were constructed by sex, age group, county, and insurance type. The dominant core chains and corresponding path lengths are summarized in Table 4, and subgroup-specific inpatient flows are presented in Figure 3.

**Table 4.** Dominant recorded comorbidity progression chains by age group, sex, county, and insurance type among inpatients with at least one recorded hypertension diagnosis across 6 hospitals in 3 counties in Henan Province, China, 2019‐2023.

Stratified factors and subgroup	Core chain (*ICD-10*^a^)	Depth (edges)	Diameter (nodes)
Age (y)			
30‐39	I10.X → I20.0	1	2
40‐49	I10.X → I25.1 → I50.9 → I20.0 → E11.9 → I69.3	5	6
50‐59	I10.X → I63.9 → I69.3 → J98.4 → E14.9 → N19.X	5	6
60‐69	I10.X → I25.1 → E11.9 → I61.5 → I69.1 → I70.8 → I63.3	6	7
≥70	I10.X → I63.9 → I50.9 → I69.3 → J98.4 → G31.9	5	6
Sex			
Male	I10.X → I63.9 → I50.9 → E11.9 → I20.0 → I25.5 → I69.3	6	7
Female	I10.X → I25.1 → I63.9 → I50.9 → J98.4 → G31.9	5	6
County			
Jia County	I10.X → I63.9 → I50.9 → E11.9 → I20.0 → J98.4 → I69.3	6	7
Yiyang County	I10.X → I25.1 → I50.9 → I63.5 → I69.3 → J98.4 → J44.9	6	7
Mianchi County	I10.X → I63.9 → I50.9 → I25.5 → I61.9 → J98.4 → I69.3	6	7
Insurance			
URRBMI^b^	I10.X → I25.1 → I63.9 → I50.9 → E11.9 → J98.4 → I69.3	6	7
UEBMI^c^	I10.X → I25.1 → I69.3 → J98.4 → K80.1 → G47.0	5	6
Other	I10.X → I63.9 → I50.9 → E11.4 → N19.X → G31.9	5	6

a *ICD-10*: *International Classification of Diseases, 10th Revision*.

b URRBMI: Urban Resident Basic Medical Insurance.

c UEBMI: Urban Employee Basic Medical Insurance.

**Figure 3.**
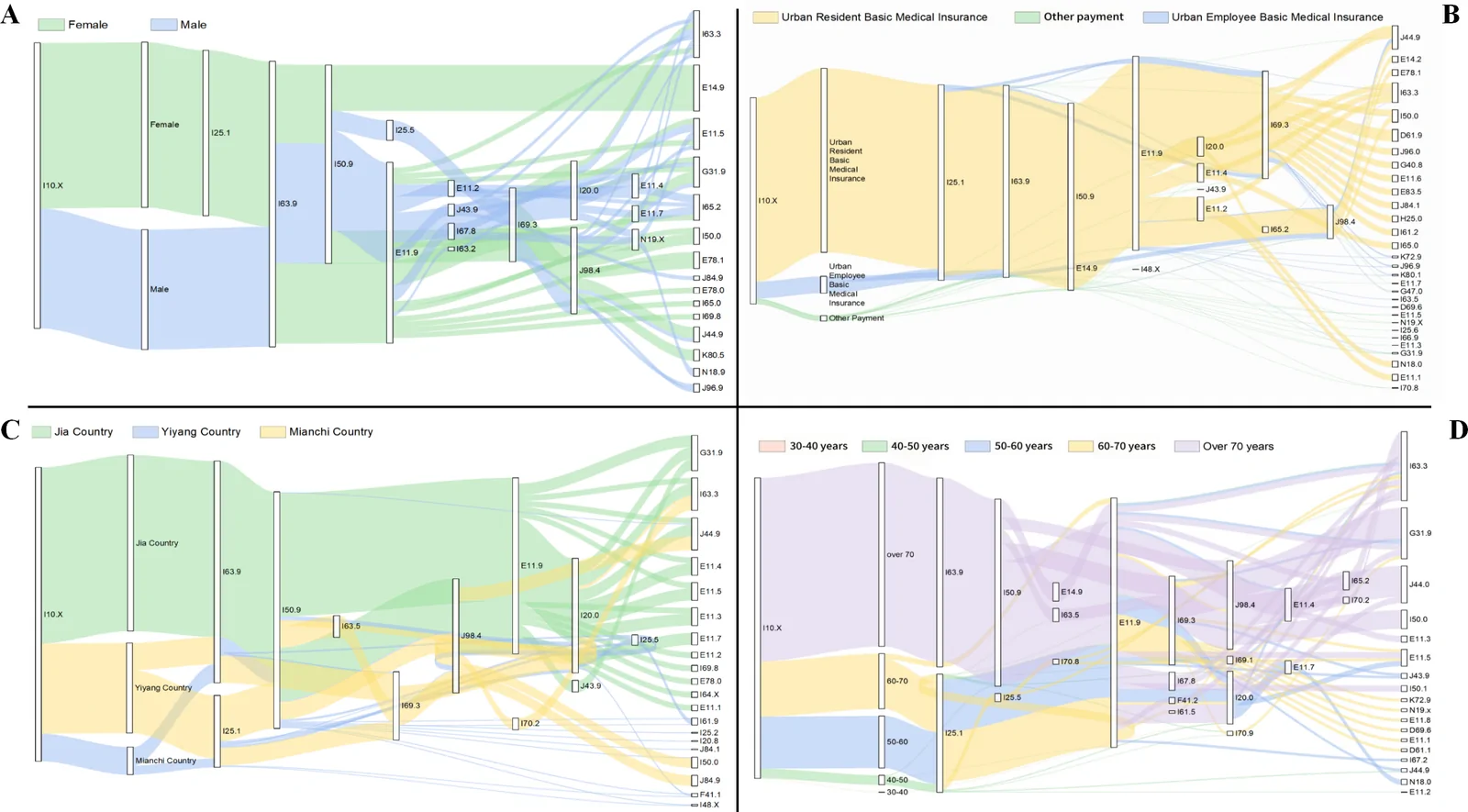
Inpatient flows in recorded comorbidity progression pathways originating from hypertension (I10.X) among 34,099 inpatients across 6 hospitals in 3 counties in Henan Province, China, 2019‐2023, stratified by sex, insurance type, county, and age group: (A) sex; (B) insurance type; (C) county; and (D) age group.

Sex-stratified networks were constructed for 17,071 male and 17,028 female inpatients. The dominant male core chain began with hypertension (I10.X) followed by cerebral infarction (I63.9), heart failure (I50.9), and type 2 diabetes (E11.9), with additional downstream cardiovascular and cerebrovascular conditions. In the female network, the dominant core chain began with hypertension, followed by atherosclerotic heart disease (I25.1), cerebral infarction, and heart failure. These results indicate that the ordering of the principal recorded cardiovascular conditions differed between the male and female networks.

Age-stratified networks were examined for inpatients aged <30 years (n=77), 30 to 39 years (n=424), 40 to 49 years (n=2101), 50 to 59 years (n=6,659), 60 to 69 years (n=9848), and ≥70 years (n=14,990). Because the subgroup aged less than 30 years contained only 77 inpatients and did not yield a sufficiently stable core chain, its pathway was not reported in Table 4. Therefore, the age-specific core chains presented below refer to inpatients aged 30 years or older.

In the 30 to 40 age group, the predominant comorbidity pathway was from hypertension to unstable angina. The 40 to 50 age group exhibited a relatively limited range of downstream recorded conditions following hypertension. Cerebral infarction (I63.9) was one of the principal downstream recorded conditions in this age group. Type 2 diabetes (E11.9) also appeared as a downstream recorded condition in this age-group network. The networks for inpatients aged 50 to 69 years included transitions involving heart failure (I50.9), Alzheimer disease (G31.9), and diabetes complications (E11.X). The network for inpatients aged ≥70 years included a broader range of recorded conditions across multiple disease systems, and it included recorded transitions involving ischemic heart disease (I20.0), chronic kidney disease (N18.9), hyperlipidemia (E78.1), and cerebrovascular disorders (I64.X).

Using health insurance type as a proxy for socioeconomic status, the study found disparities in recorded comorbidity transition patterns. The largest group, covered by the urban and rural residents’ health insurance, displayed a complex and extensive disease network with transitions to J44.9, D61.9, E14.2, and I50.0. A distinctive feature in this group was the early and common progression from hypertension to atherosclerotic heart disease (I25.1). Their recorded downstream conditions were more limited, with fewer types and quantities of downstream end-point diseases.

In terms of county distribution, inpatients from Jia County exhibited the overall trends observed in the study cohort, with a core progression pathway from hypertension (I10.X) to cerebral infarction (I63.9), followed by type 2 diabetes (E11.9) and ischemic heart disease (I20.0). Inpatients from Yiyang County exhibited a different pattern, with a more prominent intermediate disease stage of atherosclerotic heart disease (I25.1). After progressing through the pathway centered on atherosclerotic heart disease (I25.1), a higher proportion of inpatients progressed to hyperlipidemia (E78.0) and other cerebral infarction sequelae (I69.8). Inpatients from Mianchi County were characterized by their later-stage disease progression. Compared with other counties, these inpatients had a higher risk of progressing to atherosclerosis (I70.2) and cerebral infarction sequelae (I69.3) after experiencing cardiovascular and cerebrovascular events. Furthermore, the transition to emphysema (J43.9) was more prominent in the Mianchi County network than in the other county-specific networks.

## Discussion

### Principal Findings

TNA represents a substantive methodological advancement over traditional cross-sectional multimorbidity analyses by explicitly modeling directionality and the temporal ordering of diagnoses recorded across successive inpatient admissions. Most conventional multimorbidity studies have focused on static disease clusters or coprevalence matrices, which obscure the sequential nature of chronic disease accumulation [3,4]. Consistent with population-wide disease trajectory studies using Danish registry data, which demonstrated that disease pairs exhibit nonrandom and directional progression patterns over time [12], our TNA results reveal statistically significant and reproducible transition pathways rather than mere co-occurrence. In contrast to earlier hospital-based studies that relied on undirected comorbidity networks [13], our directed framework captures how diseases are temporally ordered in the available inpatient records, rather than only which conditions coexist. Notably, hypertension occupied a central upstream position in the network, with strong directed edges toward atherosclerotic heart disease and cerebral infarction. This finding corroborates large-scale temporal trajectory analyses, showing hypertension as a common upstream condition preceding ischemic heart disease and stroke [6,12]. By explicitly modeling directionality, our study extends prior work by identifying a densely interconnected temporal pattern involving hypertension, atherosclerotic heart disease, and cerebral infarction, which would remain indistinguishable in undirected network models. These directed associations reflect the temporal ordering of diagnoses in the available inpatient data and should not be interpreted as proof of causal disease progression.

Our subgroup analyses revealed pronounced sex-specific progression pathways. Male inpatients showed a more direct transition from hypertension to cerebral infarction, whereas female inpatients were more likely to progress first to atherosclerotic heart disease before subsequent cerebrovascular events. This pattern is consistent with epidemiological evidence, indicating that women experience adverse cardiovascular outcomes at lower blood pressure thresholds than men [14] and that hypertension-related vascular remodeling differs by sex [15]. Furthermore, estrogen-related mechanisms provide biological plausibility for our observed pathways. Sabbatini and Kararigas [16] demonstrated that postmenopausal estrogen decline exacerbates endothelial dysfunction, inflammation, and oxidative stress, accelerating coronary and vascular damage in women with hypertension. Our network-level finding of earlier coronary involvement among women empirically supports these mechanistic insights at the population level. In contrast to studies that report higher overall hypertension prevalence among men in early adulthood [14], our findings suggest that progression severity and pathway structure, rather than prevalence alone, differ substantially by sex—an aspect often overlooked in prior cross-sectional analyses.

Age-stratified analyses showed broader and more multisystem recorded pathways in the older age groups. Inpatients aged 40 to 49 years exhibited relatively constrained cardiovascular trajectories, whereas those aged 50 to 70 and particularly ≥70 years developed complex, multisystem disease networks involving metabolic, respiratory, and neurocognitive conditions. This observation is consistent with longitudinal evidence from the English Longitudinal Study of Aging, which showed that multimorbidity burden increases not only in prevalence but also in systemic complexity with advancing age [17]. Importantly, Bendayan et al [17] reported that specific multimorbidity patterns, including hypertension combined with diabetes and CVD, were associated with accelerated functional decline in older adults. Our findings extend this work by describing the temporal ordering of diagnoses within these patterns, rather than treating them as static states. Moreover, the prominence of diabetes as a transitional node from midlife onward aligns with MorbiNet findings, where type 2 diabetes was shown to connect CVD to multiple downstream complications despite moderate overall centrality [13]. This supports the interpretation that diabetes functions as a bridge disease within multimorbidity trajectories.

Our comparison across health insurance types revealed that the network for inpatients covered by Urban Resident Basic Medical Insurance contained a longer and more diverse dominant recorded pathway than that for inpatients covered by Urban Employee Basic Medical Insurance. The less extensive downstream patterns observed among inpatients covered by Urban Employee Basic Medical Insurance may reflect differences in population characteristics, continuity of care, preventive service use, or diagnostic opportunities across insurance groups. However, these factors were not directly measured in this study. More broadly, these insurance-related differences should be considered alongside nationwide evidence, showing that hypertensive inpatients with multimorbidity face significantly higher risks of catastrophic health expenditure, particularly among socioeconomically disadvantaged and nonemployee insurance groups [2]. Fu and Chen [2] demonstrated that multimorbidity increased the odds of catastrophic health expenditure by over 20% and that insurance protection was insufficient to offset this risk. Our temporal findings provide a disease-mechanistic explanation for these economic outcomes: more complex and accelerated multimorbidity trajectories likely translate into higher cumulative treatment costs and health care use. In contrast to studies focusing solely on expenditure outcomes [2], our work provides complementary information on the recorded disease pathways that may accompany such financial vulnerability.

County-level analyses revealed substantial heterogeneity in disease progression patterns, even within the same province. These findings are consistent with prior evidence that multimorbidity patterns vary by geographic and socioeconomic context [13,17] but extend the literature by showing that the order and speed of disease accumulation also differ across counties. For example, counties with higher progression toward peripheral vascular disease may reflect differential lifestyle exposures or health care accessibility, whereas elevated emphysema transitions following CVD may indicate environmental or occupational risks. Such place-based heterogeneity reinforces calls for localized public health strategies rather than uniform chronic disease management frameworks.

The temporal organization of the observed disease network has important implications for prevention-oriented hypertension management. The prominent transitions linking recorded hypertension with atherosclerotic heart disease, cerebral infarction, and heart failure suggest that hypertension management should be considered within a broader cardiovascular risk framework rather than focusing exclusively on blood pressure levels [18]. Rather than treating each subsequently recorded condition as an isolated event, clinicians and chronic disease management programs may benefit from considering these conditions as components of an evolving multimorbidity profile. The bridging position of type 2 diabetes between cardiovascular and multisystem disease clusters further supports integrated cardiometabolic and kidney risk assessment, including the appropriate evaluation of glycemic status and renal function [19,20]. Nevertheless, these network-defined transitions represent population-level temporal associations and should not be interpreted as deterministic or causal pathways for individual inpatients.

From a public health perspective, the heterogeneity observed across sex, age, insurance type, and county provides evidence for evaluating more risk-stratified and context-sensitive approaches to chronic disease prevention. Older inpatients, whose networks involved a broader range of metabolic, respiratory, renal, and neurological conditions, may benefit from more comprehensive, individualized care and improved coordination across clinical services [21]. County-level variation may also help identify communities in which screening, longitudinal follow-up, and integrated chronic disease services require further evaluation or prioritization. This interpretation is consistent with place-based chronic disease prevention frameworks that use local population data to inform priority setting, program planning, and resource allocation [22]. Differences across insurance groups may similarly identify populations facing greater challenges in continuity and coordination of care; however, insurance type may reflect multiple unmeasured socioeconomic and health care access factors and should not be interpreted as a causal determinant of disease progression. Therefore, the subgroup patterns identified in this study can inform local needs assessment and the development of targeted pilot strategies, but specific resource-allocation decisions would require external validation and evaluation of feasibility, effectiveness, and cost.

From a health data science perspective, this study demonstrates the value of integrating longitudinal EHR data with temporal network analytics to move beyond static descriptive analyses toward the characterization of population-level temporal patterns. Similar to prior trajectory-based frameworks [6,12], our approach enables early identification of high-risk transitions, supporting anticipatory intervention rather than reactive care. Importantly, by incorporating demographic, socioeconomic, and geographic stratification into the network analysis, our framework is consistent with precision public health approaches that use population-level data to identify groups that may benefit from more targeted and timely preventive strategies [23].

### Limitations

This study has several limitations. First, the retrospective observational design precludes causal inference. The directed edges in the TCN represent the temporal ordering of diagnoses recorded across successive inpatient admissions, rather than confirmed biological disease progression. Moreover, the earliest recorded occurrence of a condition in the study dataset may not correspond to its true onset or initial clinical diagnosis.

Second, the use of inpatient EHR data introduces selection considerations. Individuals whose hypertension and other chronic conditions were managed exclusively in outpatient or primary care settings were not captured, and people with limited access to hospital services may be underrepresented. Conversely, hospitalized patients may have greater morbidity or more intensive health care use and therefore more opportunities for additional conditions to be recorded. The observed temporal patterns should, consequently, be interpreted as patterns among inpatients with a recorded hypertension diagnosis at the participating hospitals and should not be generalized directly to the broader population of individuals with hypertension. Future studies integrating inpatient, outpatient, primary care, and community-based data would be valuable for assessing whether similar temporal patterns are observed across the broader hypertension population.

### Conclusion

This study identified structured and heterogeneous temporal patterns of multimorbidity among inpatients with recorded hypertension across 3 counties in rural Henan Province. Cardiovascular and cerebrovascular conditions formed the principal recorded progression pathways, with atherosclerotic heart disease, cerebral infarction, and heart failure occupying prominent positions in the network. Type 2 diabetes appeared to connect cardiovascular pathways with broader metabolic, renal, and other multisystem complications. Differences by sex, age, insurance type, and county further indicated that recorded multimorbidity progression was not uniform across population groups.

These findings highlight the potential value of incorporating longitudinal disease patterns into hypertension-related chronic disease prevention and management. Population-specific network patterns may help identify priorities for risk assessment, continuity of care, and locally adapted service planning. However, the observed transitions represent population-level temporal associations in inpatient records rather than causal or deterministic disease pathways, and their application to specific prevention strategies requires external validation.

## Supplementary material

10.2196/93217 Checklist 1 STROBE checklist.

10.2196/93217 Checklist 2 RECORD checklist.

## Abbreviations

CVD: cardiovascular disease
EHR: electronic health record
*ICD-10*: *International Classification of Diseases, 10th Revisio*n
RECORD: Reporting of Studies Conducted Using Observational Routinely Collected Health Data
STROBE: Strengthening the Reporting of Observational Studies in Epidemiology
TCN: temporal comorbidity network
TNA: temporal network analysis
