# Supplementary material for: Temporal Patterns of Multimorbidity Progression Among Inpatients With a Recorded Hypertension Diagnosis Across Three Counties in Rural Henan, China: Retrospective Cohort Study Using Temporal Network Analysis
Source: JMIR Public Health Surveill. 2026 Oct 23;12:e93217. doi: 10.2196/93217 (PMC13693825; doi:10.2196/93217)
Supplement: Supplementary file 1 [file publichealth-v12-e93217-s001.pdf]

Multimedia Appendix 1

STROBE Statement—Checklist of Items for Cohort Studies

Temporal Patterns of Multimorbidity Progression Among Inpatients With a Recorded Hypertension Diagnosis Across Three Counties in Rural Henan, China: A Temporal Network Analysis

Page references correspond to the 16-page highlighted revised manuscript “(Tracked changes version)_revision manuscript.docx”.

Section/topic	Item No.	Recommendation	Reported on page(s)
**Title and abstract**	1(a)	Indicate the study’s design with a commonly used term in the title or the abstract.	1–2 (analytical approach in title/abstract; “retrospective observational study” stated on p 2)
	1(b)	Provide in the abstract an informative and balanced summary of what was done and what was found.	1
**Introduction**	2	Explain the scientific background and rationale for the investigation being reported.	1–2
	3	State specific objectives, including any prespecified hypotheses.	2
**Methods**	4	Present key elements of study design early in the paper.	2
	5	Describe the setting, locations, and relevant dates, including periods of recruitment, exposure, follow-up, and data collection.	2–3
	6(a)	Give the eligibility criteria, and the sources and methods of selection of participants. Describe methods of follow-up.	3–5; 6 (Figure 1)
	6(b)	For matched studies, give matching criteria and number of exposed and unexposed.	Not applicable (unmatched cohort)
	7	Clearly define all outcomes, exposures, predictors, potential confounders, and effect modifiers. Give diagnostic criteria, if applicable.	2–5; 6–11
	8*	For each variable of interest, give sources of data and details of methods of assessment (measurement). Describe comparability of assessment methods if there is more than one group.	2–5
	9	Describe any efforts to address potential sources of bias.	3–5; 14
	10	Explain how the study size was arrived at.	3–4; 6 (all eligible records; no a priori sample-size calculation)

Section/topic	Item No.	Recommendation	Reported on page(s)
	11	Explain how quantitative variables were handled in the analyses. If applicable, describe which groupings were chosen and why.	4–5; 6–11
	12(a)	Describe all statistical methods, including those used to control for confounding.	4–5 (network tests and robustness analyses; no confounder-adjusted model)
	12(b)	Describe any methods used to examine subgroups and interactions.	1; 10–11 (subgroup-specific networks; no formal interaction tests)
	12(c)	Explain how missing data were addressed.	3
	12(d)	If applicable, explain how loss to follow-up was addressed.	Not applicable (retrospective EHR cohort; no prospective loss to follow-up)
	12(e)	Describe any sensitivity analyses.	5; 8–9
**Results**	13(a)	Report numbers of individuals at each stage of study—for example, numbers potentially eligible, examined for eligibility, confirmed eligible, included in the study, completing follow-up, and analysed.	3–4; 5–6
	13(b)	Give reasons for non-participation at each stage.	3–4; 6 (Figure 1)
	13(c)	Consider use of a flow diagram.	6 (Figure 1)
	14(a)	Give characteristics of study participants (for example, demographic, clinical, social) and information on exposures and potential confounders.	6–7 (Table 1)
	14(b)	Indicate number of participants with missing data for each variable of interest.	3 (4,300 records excluded for missing key fields; variable-specific missingness not reported)
	14(c)	Summarise follow-up time (for example, average and total amount).	7
	15*	Report numbers of outcome events or summary measures over time.	7–11
	16(a)	Give unadjusted estimates and, if applicable, confounder-adjusted estimates and their precision. Make clear which confounders were adjusted for and why they were included.	8–11 (unadjusted network measures and transition summaries; no adjusted estimates)
	16(b)	Report category boundaries when continuous variables were categorized.	6–7; 10–11
	16(c)	If relevant, consider translating estimates of relative risk into absolute risk for a meaningful time period.	Not applicable (no relative-risk estimates)

Section/topic	Item No.	Recommendation	Reported on page(s)
	17	Report other analyses done—for example, analyses of subgroups and interactions, and sensitivity analyses.	8–11
**Discussion**	18	Summarise key results with reference to study objectives.	12–15
	19	Discuss limitations of the study, taking into account sources of potential bias or imprecision. Discuss both direction and magnitude of any potential bias.	14 (sources and implications of bias discussed; magnitude not quantified)
	20	Give a cautious overall interpretation of results considering objectives, limitations, multiplicity of analyses, results from similar studies, and other relevant evidence.	12–15
	21	Discuss the generalisability (external validity) of the study results.	14
**Other information**	22	Give the source of funding and the role of the funders for the present study and, if applicable, for the original study on which the present article is based.	15

Notes: Page locations were checked against the final 16-page highlighted revised manuscript. Because the manuscript does not contain continuous line numbering, pages and relevant sections, tables, or figures are listed. “Not applicable” is used where the item does not apply to this unmatched retrospective EHR cohort. Where an item is only partly reported, the limitation is stated in the page-reference cell.

Checklist source: STROBE cohort checklist (STROBE Initiative).
