# Supplementary material for: Temporal Patterns of Multimorbidity Progression Among Inpatients With a Recorded Hypertension Diagnosis Across Three Counties in Rural Henan, China: Retrospective Cohort Study Using Temporal Network Analysis
Source: JMIR Public Health Surveill. 2026 Oct 23;12:e93217. doi: 10.2196/93217 (PMC13693825; doi:10.2196/93217)
Supplement: Supplementary file 2 [file publichealth-v12-e93217-s002.pdf]

Multimedia Appendix 2

RECORD Statement—Checklist for Studies Using Routinely Collected Health Data

Temporal Patterns of Multimorbidity Progression Among Inpatients With a Recorded Hypertension Diagnosis Across Three Counties in Rural Henan, China: A Temporal Network Analysis

Page references correspond to the 16-page highlighted revised manuscript “(Tracked changes version)_revision manuscript.docx”.

Section/topic	Item No.	RECORD recommendation	Reported on page(s)	Manuscript-specific note
**Title and abstract**	1.1	The type of data used should be named in the title or abstract. Where possible, the names of the databases used should be included.	1	Electronic health record (EHR) data are identified in the abstract. The participating hospitals and data source are described in the Methods; no formal database name is reported.
	1.2	If applicable, the geographic region and time frame within which the study took place should be reported in the title or abstract.	1	The title/abstract report three rural counties in Henan, China, and the 2019–2023 study period.
	1.3	If linkage between databases was conducted for the study, this should be clearly stated in the title or abstract.	Not applicable	No linkage across two or more databases was conducted. Repeated admissions were linked within the same EHR dataset.
**Methods—Participants**	6.1	The methods of study population selection (such as codes or algorithms used to identify subjects) should be listed in detail. If this is not possible, an explanation should be provided.	3–4; 6 (Figure 1)	Eligibility criteria, the ICD-10 hypertension code (I10.x), record exclusions, preprocessing rules, and the cohort-selection flow diagram are reported.
	6.2	Any validation studies of the codes or algorithms used to select the population should be referenced. If validation was conducted for this study and not published elsewhere, detailed methods and results should be provided.	Not reported; see p 14	No published or study-specific validation of the population-selection codes/algorithm is reported. Possible ICD-10 coding variation and misclassification are acknowledged in Limitations.
	6.3	If the study involved linkage of databases, consider use of a flow diagram or other graphical display to demonstrate the data linkage process, including the number of individuals with linked data at each stage.	Not applicable	No cross-database linkage was conducted.

Section/topic	Item No.	RECORD recommendation	Reported on page(s)	Manuscript-specific note
**Methods—Variables**	7.1	A complete list of codes and algorithms used to classify exposures, outcomes, confounders, and effect modifiers should be provided. If these codes or algorithms cannot be reported, an explanation should be provided.	3–5; 7–11 (partially reported)	The ICD-10 standard, hypertension code, network-construction rules, and principal disease codes are reported. A complete list of all disease codes/nodes is not provided in the manuscript.
**Methods—Statistical methods**	12.1	Authors should describe the extent to which the investigators had access to the database used to create the study population.	3; 15	The source-data handling and de-identification process are described in Ethical Considerations. Data-access restrictions and availability of analytic code are described in Data Availability.
	12.2	Authors should provide information on the data cleaning methods used in the study.	3	Identifier harmonization, ICD-10 standardization, date normalization, exclusion of duplicates and records missing key fields, and the decision not to impute missing diagnostic or temporal data are described.
	12.3	State whether the study included person-level, institutional-level, or other data linkage across two or more databases. Linkage techniques and methods used to evaluate linkage quality should be provided.	3–4	No linkage across separate databases was conducted. Encrypted identifiers were used for person-level linkage of multiple admissions within the same source EHR dataset.
**Results—Participants**	13.1	Describe in detail the selection of the persons included in the study, including filtering based on data quality, data availability, and linkage. The selection can be described in the text and/or by means of a study flow diagram.	3–4; 5–6 (Figure 1)	The derivation of the 34,099-inpatient cohort, data-quality exclusions, eligibility filters, and numbers retained at each stage are reported in text and Figure 1.
**Discussion—Limitations**	19.1	Discuss the implications of using data that were not created or collected to answer the specific research question(s). Include discussion of misclassification bias, unmeasured confounding, missing data, and changing eligibility over time, as they pertain to the study.	14	The Limitations section discusses the consequences of retrospective inpatient EHR data, first-recorded diagnoses rather than true onset, selection toward repeatedly hospitalized inpatients, ICD-10 coding variation/misclassification, unavailable clinical/lifestyle/socioeconomic variables, and limited generalizability.

Section/topic	Item No.	RECORD recommendation	Reported on page(s)	Manuscript-specific note
**Other information**	22.1	Authors should provide information on how to access any supplemental information such as the study protocol, raw data, or programming code.	3; 15	The STROBE and RECORD checklists are identified as Multimedia Appendices 1 and 2. Raw data are not publicly available; access requires data-custodian approval, and analytic code is available from the corresponding authors on reasonable request.

Notes: RECORD extends STROBE for observational studies using routinely collected health data. Page locations were checked against the final 16-page highlighted revised manuscript. Because the manuscript does not contain continuous line numbering, pages and relevant sections, tables, or figures are listed. Where a RECORD item is not applicable or is only partly reported, this is stated explicitly.

Checklist source: Benchimol EI, et al. The REporting of studies Conducted using Observational Routinely-collected health Data (RECORD) Statement. PLoS Med. 2015;12(10):e1001885.
